# Necrolytic migratory erythema is an important visual cutaneous clue of glucagonoma

**DOI:** 10.1038/s41598-022-12882-2

**Published:** 2022-05-31

**Authors:** Wei Li, Xue Yang, Yuan Deng, Yina Jiang, Guiping Xu, Enxiao Li, Yinying Wu, Juan Ren, Zhenhua Ma, Shunbin Dong, Liang Han, Qingyong Ma, Zheng Wu, Zheng Wang

**Affiliations:** 1 Department of Hepatobiliary Surgery, The First Affiliated Hospital of Xi’an Jiaotong University, Xi’an, Shaanxi 710061 People’s Republic of China; 2 Department of Pathology, The First Affiliated Hospital of Xi’an Jiaotong University, Xi’an, Shaanxi 710061 People’s Republic of China; 3 Department of Radiology, The First Affiliated Hospital of Xi’an Jiaotong University, Xi’an, Shaanxi 710061 People’s Republic of China; 4 Department of Medical Oncology, The First Affiliated Hospital of Xi’an Jiaotong University, Xi’an, Shaanxi 710061 People’s Republic of China; 5 Department of Radiotherapy Oncology, The First Affiliated Hospital of Xi’an Jiaotong University, Xi’an, Shaanxi 710061 People’s Republic of China

**Keywords:** Cancer, Endocrine cancer

## Abstract

Glucagonoma is an extremely rare neuroendocrine tumor that arises from pancreatic islet alpha cells. Although glucagonoma is usually accompanied by a variety of characteristic clinical symptoms, early diagnosis is still difficult due to the scarcity of the disease. In this study, we present the cumulative experiences, clinical characteristics and treatments of seven patients diagnosed with glucagonoma during the past 10 years at the First Affiliated Hospital of Xi’an Jiaotong University. The seven patients in our cohort consisted of six females and one male with an average diagnosis age of 40.1 years (range 23–51). The average time from onset of symptoms to diagnosis of glucagonoma was 14 months (range 2–36 months). All the patients visited dermatology first for necrolytic migratory erythema (NME) 7/7 (100%), and other presenting symptoms included diabetes mellitus (DM) 4/7 (57%), stomatitis 2/7 (28%), weight loss 4/7 (57%), anemia 4/7 (57%), diarrhea 1/7 (14%), and DVT1/7 (14%). Plasma glucagon levels were increased in all patients (range 216.92–3155 pg/mL) and declined after surgery. Imaging studies revealed that four of seven patients had liver metastasis. Six of seven patients received surgical resection, and all of them received somatostatin analog therapy. Symptoms improved significantly in 6 out of 7 patients. Three of seven patients died of this disease by the time of follow-up. Our data suggest that if persistent NME is associated with DM and high glucagon levels, timely abdominal imaging should be performed to confirm glucagonoma. Once diagnosed, surgery and somatostatin analogs are effective for symptom relief and tumor control.

## Introduction

Glucagonoma is an extremely rare and slow-growing functional pancreatic neuroendocrine tumor arising from islet alpha cells in the tail of the pancreas. It usually presents with glucagonoma syndrome associated with characteristic clinical symptoms, including necrolytic migratory erythema (NME), diabetes mellitus (DM), stomatitis, anemia, deep vein thrombosis (DVT), weight loss, diarrhea and other symptoms^1^. With the exception of NME, other clinical manifestations are nonspecific, which accounts for the delay in diagnosis in most cases and also for the fact that at least 50% of cases already have metastatic disease at the time of diagnosis.

NME is observed in approximately 70–90% of patients diagnosed with glucagonoma^2,3^. This rash is usually widespread, and the major sites of involvement are the perioral region, trunk, extremities and perineum^4,5^. The distinguishing feature of NME is annular erythematous plaques with central bullous, ulcerative lesions surrounded by brown pigment, which are usually pruritic and painful^5,6^. The histological features of this skin lesion include parakeratosis, hyperkeratosis, spongiosis of the epidermis with necrolysis, loss of the granular layer, vacuolization of keratinocytes, and perivascular and interstitial inflammation^7^.

This paper summarizes the clinical characteristics of seven typical patients with glucagonoma followed at our hospital during the past 10 years. Our cumulative experiences (including diagnosis and treatment) may help clinicians to better recognize, diagnose and treat glucagonoma.

## Patients and methods

This study was approved by the Ethics Committee of the First Affiliated Hospital of Xi’an Jiaotong University and the study was conducted in accordance with the approved guidelines. Informed consent was obtained from all subjects and/or their legal guardian(s). We reviewed the database and collected seven cases of glucagonoma in the past 10 years. Patients with clinical presentations of skin manifestation (the skin rash is characterized by an intense erythematous lesion, which shows superficial epidermal necrosis and spreads in a centrifugal pattern), glucagonoma syndrome, elevated plasma glucagon, and a pathological diagnosis of pancreatic islet cell tumor were included in this cohort. The medical records of the included patients were reviewed. Tumor diameters were obtained from CT scan measurements. Follow-up data, including patients’ follow-up status, symptoms (skin rash), recovery and administration of other therapies, were acquired from hospital medical records or by phone interviews with the patients, relatives, or general practitioners.

## Results

### Case presentation

The seven patients consisted of six females and one male, and the median age at diagnosis was 40.1 years (range 23–51) (Table 1). The duration from initial symptom presentation to final diagnosis ranged from 2 months to 3 years (Table 1). The most common symptoms of all seven patients with glucagonoma in the present study are presented in Table 1.

**Table 1:** Patient presentation, tumor Characteristics and treatments.

Patient	1	2	3	4	5	6	7
Male/female	F	F	F	F	F	F	M
Age at diagnosis (years)	25	44	47	49	51	23	42
Symptoms at diagnosis							
NME	Y^a^	Y^a^	Y^a^	Y^a^	Y^a^	Y^a^	Y^a^
The localization where skin lesions first appear	Lower limbs	Lower limbs and face	Lower limbs and feet	Lower limbs, abdomen and dorsum	Lower limbs and ankles	Lower limbs and perioral regions	Lower limbs and gluteal regions
Diabetes	N (but with IGT)	N (but with IGT)	Y	Y	Y	Y	Y
Stomatitis (glossitis and cheilitis included)	N	N	N	Y^a^	N	Y	N
Weight loss	N	N	Y	Y	Y	N	Y
Anemia	N	N	Y	Y	Y	Y	N
Diarrhea	N	N	Y	N	N	N	N
DVT	N	N	N	Y	N	N	N
The first department visited	Dermatology	Dermatology	Dermatology	Dermatology	Dermatology	Dermatology	Dermatology
Biochemical diagnosis and disease extent							
Was skin biopsy diagnostic?	Y	Y	Y	Y	Y	Y	Y
Plasma glucagon (pg/mL)	217	720	3155	1720	920	> 800	269
Glucagon immunostain	+	+	ND	+	+	+	+
Ki-67	8%	10%	10%	NA	1. 1% 2. 8%	30%	8%
CgA (ng/mL)	+	+	ND	+	1. - 2. +	+	+
NSE (ng/mL)	43.05	+	29.65	+	19.54	Normal	+
SSTR2	+	–	ND	–	1. – 2. ±	+	+
Gastrin (pg/mL)	ND	–	ND	–	1. – 2. ±	–	–
Syn	+	+	ND	+	1. - 2. +	+	+
ESR	ND	ND	ND	ND	ND	ND	ND
Time from symptoms to diagnosis (months)	8	3	2	24	12	14	36
Pancreatic tumor location	Head	Head	Tail	Tail	Body and tail	Head	Head
Tumor size (cm)	3.1 × 2.3 × 1.6	3.5 × 2.1 × 2.0	2.3 × 2.0 × 2.0	8.0 × 5.0 × 3.5	6.5 × 5.0 × 4.5	4.2 × 6.0 × 5.0	4.2 × 5.0 × 4.7
Location of metastasis	N	N	Liver	Liver, lymph nodes	Liver, lymph nodes	Liver	N
TNM staging	IIa (T2, N0, M0)	IIa (T2, N0,M0)	IV (T2, Nx, M1)	IV (T3, N1, M1)	IV (T3, N1, M1)	IV (T3, N0, M1)	IIb (T3, N0, M0)
Surgery	R-PPPD	PD	ND	DP + splenectomy	1. LDP + splenectomy + liver nodule biopsy resection 2. Open exploration + adhesionolysis + pancreatectomy	PD + hepatic metastasectomy	PD
Somatostatin analogue	Octreotide LAR 30 mg × 1/month	Octreotide 100 μg × 3/day	Octreotide LAR 30 mg × 1/month	Octreotide 600 μg/day	Octreotide LAR 30 mg × 1/month	Octreotide LAR 30 mg × 1/month	Octreotide 100 μg × 3/day
Amino acid infusions	Y	Y	Y	Y	Y	Y	Y
Chemotherapy	N	N	N	N	Sulfatinib; Everolimus	N	N
PRRT	N	N	N	90Y-DOTALAN 200 mCi × 2	N	N	N
Other treatments	N	N	N	TACE, RFA	TACE	N	N
Glucagon after surgery (pg/mL)	103	233	ND	816	ND	315	110
Symptoms improved	3 days after surgery	1 week after surgery	N	Y	1 day after surgery	3 days after surgery	1 day after surgery
Survival (months)	Alive	Alive (> 5 years)	11 ( diagnosis) 13 (first symptom)	63 (diagnosis) 78 (first symptom)	156 (diagnosis) 168 (first symptom)	Alive	Alive

Normal glucagon levels < 200 pg/mL.

*NME* Necrotizing migratory erythema, *DVT* Deep vein thrombosis, *IGT* Impaired glucose tolerance, *R-PPPD* Robotassisted pylorus-preserving pancreaticoduodenectomy, *DP* Distal pancreatectomy, *PD* Pancreaticoduodenectomy, *TACE* Transarterial chemoembolization, *PRRT* Peptide receptor radioligand therapy, *RFA* Radiofrequency ablation, *N* No, *Y* Yes, *ND* Not done, *NA* Not available.

^a^First arose symptom.

**Figure 1:**
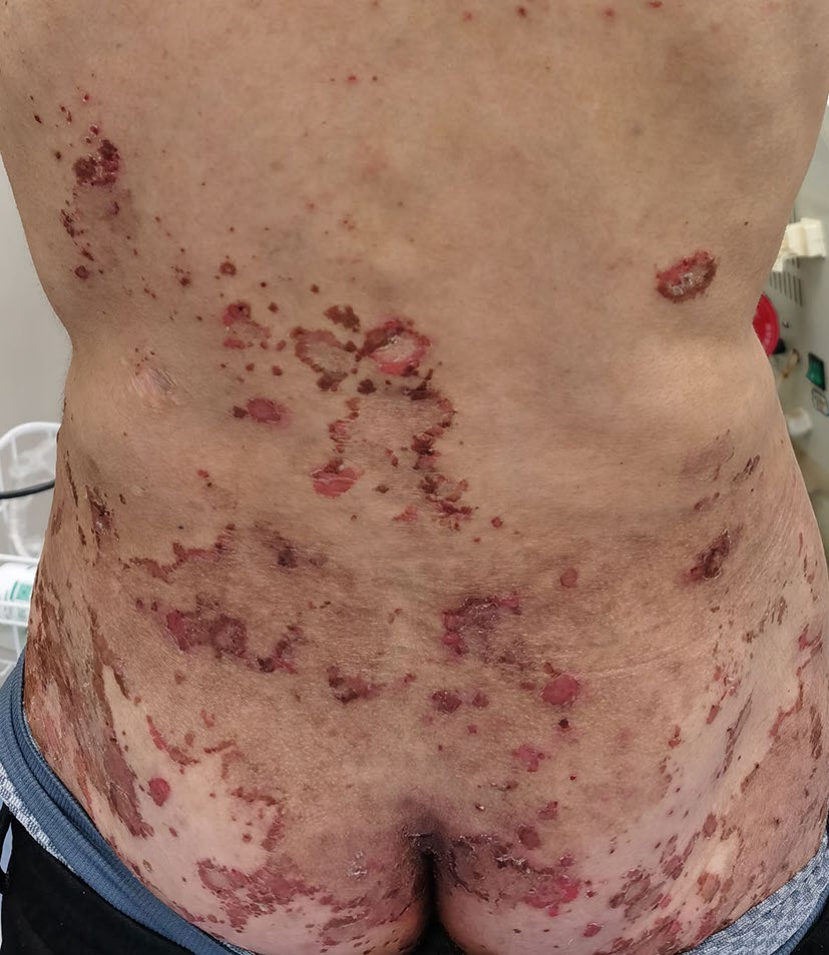
NME of glucagonoma. Skin lesions affecting lower back and gluteal regions. The boundary of the rash is not clear. Erosion, exudation and necrosis can be seen in the center of some erythematous lesions.

NME was the first symptom in all patients. Patient 4 presented with repeated stomatitis, and atrophic glossitis accompanied by NME as the first symptom. Patient 6 complained of NME as the first symptom, and then symptoms such as stomatitis and glossitis developed. All the patients reported in this study had a delayed diagnosis. All the patients (7/7) visited the dermatology department first with a complaint of pruritic and painful polymorphic rash with a duration of several months. The rash initially appeared at different places, such as the extremities, faces, ankles, gluteal regions, perineum, groins, lower back or abdomens. Then, the erythema slowly spread throughout the body. The boundary of the rash was not clear, and some of the lesions were fused in large areas. Erosion, exudation and necrosis can be seen in the center of some erythematous lesions. Figure 1 showed a typical image of NME in lower back and gluteal regions (Fig. 1). Topical steroid treatment led to a temporary improvement of the erythema in 6 of 7 patients, but it still occurred repeatedly, and the erythema reoccurred accompanied by even much more severe pruritus and pain. There was only one patient who was treated with topical steroids but did not have any clinical improvement.

While not all of the patients initially had diabetes, all of the patients eventually developed diabetes (5/7) or IGT (2/7). Other symptoms included stomatitis and glossitis (2/7), weight loss (4/7), anemia (4/7), diarrhea (1/7), and DVT (1/7) (Table 1). Among these symptoms, NME and diabetes or IGT were present in all the patients and may be considered as tumor-specific symptoms.

### Examinations and disease diagnosis

**Figure 2:**
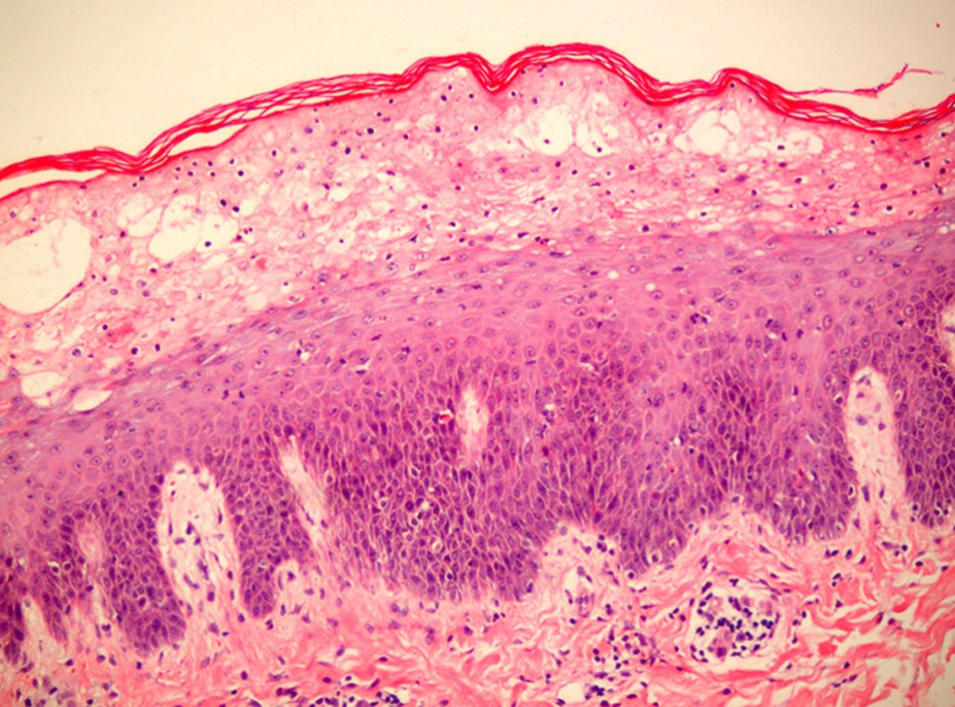
Histopathological image of skin biopsy of NME. The H&E image of the skin biopsy of NME showed mild hyperplasia of the epidermis and abundant lymphocyte infiltration (× 40).

Skin biopsies were performed in all seven cases, and skin biopsies were taken from active borders of the lesions. NME was diagnosed by the pathologist in all cases. The characteristics of the histopathological assessment included mild hyperplasia of the epidermis, hypertrophy of the spinous layer, hyperkeratosis, incomplete keratosis and local abscess formation in the superficial layer of acanthocytes, dilatation of blood vessels in the dermal papilla, and abundant lymphocyte infiltration around blood vessels (Fig. 2). Serum plasma glucagon levels were significantly elevated (range 217–3155 pg/mL; normal < 200 pg/mL) in all patients before surgery. Other biochemical abnormalities are outlined in Table 1.

Upon diagnosis, the primary tumors were identified by abdominal enhanced computed tomography (CT) in 4 of 7 cases and by magnetic resonance imaging (MRI) in 3 of 7 cases in our patient cohort. Other modalities used for diagnosis were ultrasonography (3/7), liver metastasis biopsy (2/7), laparoscopic pancreatic biopsy (3/6) and skin biopsy (7/7). All patients had histopathological confirmation of a neuroendocrine tumor.

Four tumors were located in the head of the pancreas, two tumors were located in the tail, and one was located in the body and tail of the pancreas. Four patients presented with hepatic metastases upon diagnosis, and two cases had liver and lymph node metastases. The initial tumor stage, size, sites of metastasis, pathologic characteristics and proliferation index are detailed in Table 1. Other lymph node metastatic foci were found in two patients (patients 5 and 6) (Table 1).

### Therapy

The available treatment modalities used in our patients and the overall response to treatment are presented in Table 1. Six of seven cases received surgical resection (5 in our hospital, 1 in another hospital), involving pancreaticoduodenectomy (PD) (4/6) and distal pancreatectomy (DP) (2/6). Of the 4 patients who received PD and 2 patients who received DP, 1 patient each also received combined hepatic metastasectomy (Table 1). For all the 6 patients who received surgery, the skin lesions improved gradually within one week (1 day ~ 1 week) after the surgery, and postoperative plasma glucagon levels sharply decreased or even returned to normal levels. One patient (patient 3) did not undergo surgical resection and was only treated with somatostatin analogs because multiple liver metastases had been found at diagnosis. All 7 patients received somatostatin analog and amino acid infusions. Four of seven patients received octreotide LAR, and the other 3 of 7 patients received octreotide treatment. Patient 4 also underwent transarterial chemoembolization (TACE), radiofrequency ablation (RFA) and 90Y-DOTALAN due to liver metastasis. Patient 5 received chemotherapy (sulfatinib; everolimus) and TACE treatment.

### Pathological characteristics

**Figure 3:**
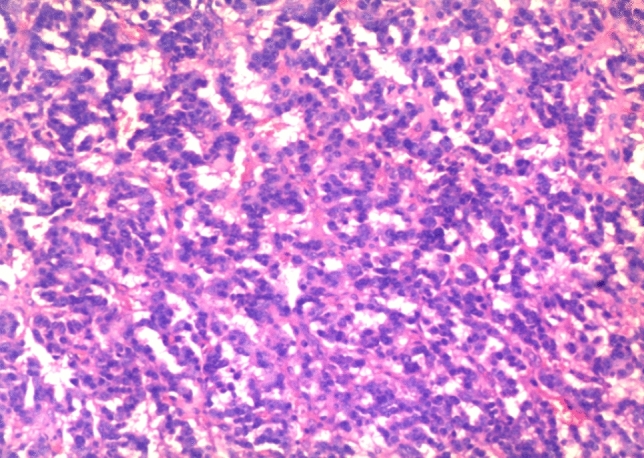
Histopathological (H&E) image of glucagonoma. The pathological examination of excised tissue showed a grade 2 pancreatic neuroendocrine tumor, with a mitotic count of 3 per 10 high-power fields (× 100).

**Figure 4:**
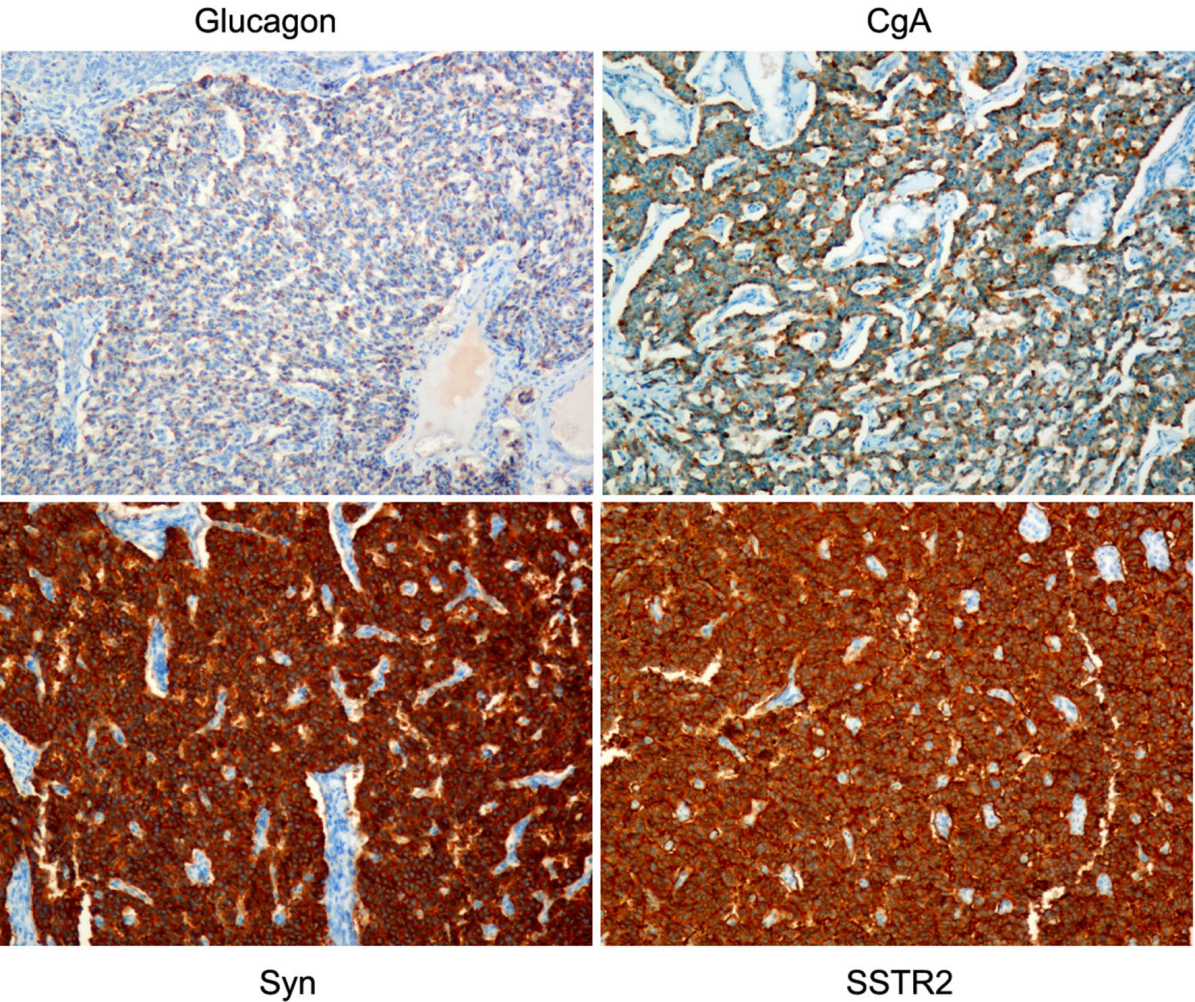
Positive immunohistochemical staining for glucagon, Cga, Syn and SSTR2 (× 100).

The primary tumor size ranged from 3.1 × 2.3 × 1.6 cm to 8.0 × 5.0 × 3.5 cm. Metastases were found in three of the six patients during surgery. Pathologists diagnosed patient 4 with poorly differentiated pancreatic islet cell tumors and other diagnosed patients with moderately differentiated pancreatic islet cell tumors. Immunohistochemistry confirmed the final diagnosis of glucagonoma. Figure 3 shows a typical histopathological image of glucagonoma. The pathological examination of excised tissue showed a grade 2 pancreatic neuroendocrine tumor, with a mitotic count of 3 per 10 high-power fields. The Ki-67 index was 8–10% for patients 1, 2, 3, 5 and 7 and 30% for patient 6 and was not tested in patient 4. Other immunohistochemical staining results for chromogranin A (CgA), synaptophysin (Syn) and somatostatin receptor 2 (SSTR2) were also analyzed (Fig. 4). In summary, 5 patients had had a grade 2 (G2) tumor, and patient 6 had a grade 3 (G3) tumor.

### Follow-up

Six patients’ (6/7) symptoms (skin rash) improved gradually within one week after surgery. Long-acting somatostatin was used after surgery, and the skin rash did not recur. Only one patient (patient 3) did not receive surgery, so his or her skin rash was not relieved. By the time of follow-up in October 2021, three out of seven patients had died, and all the deaths were disease related. The mean time to disease-related death was 6.4 years (range 0.9–13) from diagnosis and 7.2 years (range 1.1–14) from initial clinical manifestations.

## Discussion

Neuroendocrine tumors of the pancreas (pNETs) are rare, with an annual incidence of less than 0.8/100,000^8^. They include both functioning and nonfunctioning pNETs and are mostly slow-growing with a capacity to metastasize. The functioning pNETs constitute approximately 30–40% of all pNETs displaying different clinical syndromes due to hormone oversecretion by the tumor, such as excess gastrin (gastrinoma, Zollinger-Ellison syndrome), insulin (insulinoma), glucagon (glucagonoma), somatostatin (somatostatinoma) and vasoactive intestinal peptide (VIPoma). The symptoms of nonfunctioning pNETs are mainly secondary to the local mass effect of the tumor, such as abdominal pain, jaundice, and weight loss^9^.

Glucagonomas are extremely rare pNETs, with an estimated global incidence of one in 20 million people^7^. The average age at diagnosis for glucagonoma is 53.5 years, affecting men and women in almost equal proportions^7,10^. However, all the patients in our cohort were female, with an average age of 42.5 years. Most tumors are sporadic, while less than 3% of tumors are associated with multiple endocrine neoplasia type 1 (MEN1), one of the most common familial cancer syndromes^11^. In approximately 87% of cases, the glucagonoma is located in the tail of the pancreas. Over 50% are metastatic at the time of diagnosis^12^, which highlights the importance of early diagnosis of the disease.

The classic features of glucagonoma syndrome include a characteristic rash named NME, IGT or DM, DVT, depression, anemia, weight loss, hypoaminoacidemia and low zinc levels^1,13,14^. NME is usually the first clinical manifestation of glucagonoma syndrome, which often starts as pruritic and painful erythema and gradually enlarges and coalesces to form bullous lesions^15^. The pathogenesis of NME is still uncertain. Hyperglucagonemia might play an important role, as surgical removal of glucagonomas or stabilizing glucagon levels using somatostatin analogs results in rash control^7,16^. Other theories, including hypoaminoacidaemia-induced epidermal protein and micronutrient depletion and deficiency of essential fatty acids and zinc, should also be considered because nutritional support therapy and topical zinc therapy have been used to ameliorate NME^17,18^.

Surgical removal is considered to be the only definitive and curative treatment for pancreatic glucagonoma and NME^7^. Optional operations included simple enucleation (< 2 cm) with peripancreatic lymph dissection, pancreaticoduodenectomy with peripancreatic lymph dissection, distal pancreatectomy with peripancreatic lymph dissection and splenectomy. However, more than half of all glucagonomas present with metastatic disease, most commonly liver metastasis. It has been reported that synchronous resection of pancreatic neuroendocrine tumors and liver metastasis (more than 30% of the liver tissue retained) provides a more favorable outcome^19^. Liver transplantation may be considered as a potential therapeutic approach for unresectable hepatic metastases arising from pancreatic glucagonoma^20^. TACE might also be a safe therapeutic approach for liver metastasis arising from pNETs because of the highly vascular and blood supply that primarily derives from the hepatic artery^21^. In addition, RFA is usually performed in combination with surgery, which has certain advantages in removing isolated metastases^22^. Medical therapy for glucagonoma, including chemotherapeutics, somatostatin analogs, PRRT and molecular targeted drugs, are also effective in controlling clinical symptoms and tumor growth^7,16^.

In conclusion, glucagonoma is a rare type of functional pNET. Since NME might be the only clue for the early detection of this tumor, it is very important to correctly diagnose NME in a timely manner Currently, surgical intervention is the only definitive treatment for this disease. Medical therapy is effective for symptom control and metastatic disease management.

## Data Availability

The datasets used and/or analyzed during the current study are available from the corresponding author on reasonable request.
